# A High-Sensitivity Tunable Two-Beam Fiber-Coupled High-Density Magnetometer with Laser Heating

**DOI:** 10.3390/s16101691

**Published:** 2016-10-13

**Authors:** Igor Savukov, Malcolm G. Boshier

**Affiliations:** Physics Division, Los Alamos National Laboratory, Los Alamos, NM 87545, USA; boshier@lanl.gov

**Keywords:** atomic magnetometers, low-frequency, high sensitivity

## Abstract

Atomic magnetometers (AM) are finding many applications in biomagnetism, national security, industry, and science. Fiber-coupled (FC) designs promise to make them compact and flexible for operation. Most FC designs are based on a single-beam configuration or electrical heating. Here, we demonstrate a two-beam FC AM with laser heating that has 5 fT/Hz^1/2^ sensitivity at low frequency (50 Hz), which is higher than that of other fiber-coupled magnetometers and can be improved to the sub-femtotesla level. This magnetometer is widely tunable from DC to very high frequencies (as high as 100 MHz; the only issue might be the application of a suitable uniform and stable bias field) with a sensitivity under 10 fT/Hz^1/2^ and can be used for magneto-encephalography (MEG), magneto-cardiography (MCG), underground communication, ultra-low MRI/NMR, NQR detection, and other applications.

## 1. Introduction

Sensitive magnetic-field measurements are critical for many applications in science, national security, industry, and medical diagnostics. Superconducting quantum interference devices (SQUIDs) have been traditionally used in sensitive applications at low frequency, for example, in magneto-encephalography (MEG), but they require a cryogenic infrastructure. Atomic magnetometers (AMs) operating in the spin-exchange-relaxation-free (SERF) regime have demonstrated similar sensitivity to SQUIDs [1,2] and are a promising cryogen-free alternative to SQUIDs for many applications, such as MEG [3,4,5,6,7], magneto-cardiography (MCG) [8,9], ultra-low-field NMR [10,11,12], and MRI [13,14].

The first SERF magnetometers were bulky and expensive, requiring set up on an optical table [15]. Recently, however, SERF and other sensitive atomic magnetometers have been developed that are compact, flexible, and inexpensive to assemble, especially those based on a fiber-coupled (FC) design with small cells [4,5,16] or a microfabrication design [6]. Atomic magnetometers of the FC design have sufficiently matured for commercialization [16]. One driving force for the development has been MEG, with MEG recordings already demonstrated by several groups [4,5,6].

Many FC AMs have demonstrated similar sensitivities but have many different features with advantages and disadvantages important to consider in applications: the number and types of lasers, optical configurations, the size of the cell, the minimal stand-off distance, the cell composition, the temperature of operation, demonstrated and potential sensitivities, bandwidth, tunability, and operation with or without field modulation. Shah and Wakai [5] presented MEG and MCG recordings with an FC AM of 10 fT/Hz^1/2^ sensitivity containing a 4-mm ^87^Rb Pyrex cell. The AM has a single beam configuration, with a magnetic field modulated at a kHz frequency to enable field sensitivity along the axis perpendicular to the beam direction and suppress 1/f noise. The cell was heated to 150 °C via light from a fiber-coupled laser diode. This heating method has been developed for small cells (4 mm). The laser power required increases linearly with the surface area or quadratically with the size of the cell; for large cells, there could be a problem due to the burning of the fiber attached to the cell. Johnson et al. [4] presented MEG recordings with a two-color collinear beam SERF using the D2 line to probe and the D1 to pump the 1 × 1 × 5 cm^3^ ^87^Rb cell filled with 50 Torr of N_2_ and 760 Torr of He. The cell was electrically heated with a 20 kHz ac current to temperatures of 140–180 °C. A modulated field at 1 kHz was also used to select the field sensitivity axis and reduce 1/f noise. A sensitivity of 5 fT/Hz^1/2^ was achieved. There are also demonstrations of sensitive operation with micro-fabricated AMs. When external lasers in orthogonal configurations were used with a micro-fabricated cell, a sensitivity of 5 fT/Hz^1/2^ was achieved by heating the Rb cell to 200 °C via passing an electric current through two indium-tin-oxide (ITO) windows [17]. While this result is quite impressive, several drawbacks are present: the temperature is very high, which can lead to faster cell deterioration; the operation has a 50% duty cycle due to the need to turn off the heating current during measurements; and the noise from Rb, the ITO film, and the heater connections are significant, limiting the potential for sensitivity improvement.

## 2. Materials and Methods

Here, we present a two-beam Rb-87 FC AM that relies on light heating, which is guided to the cell through a multi-mode fiber. Our sensitivity at a low frequency in the 20–80 Hz range is better than the sensitivity of other two-beam FC AMs [18,19,20] of similar design but a different method of cell heating. In particular, in [18], the demonstrated sensitivity at 20 Hz is 20 fT/Hz^1/2^; at frequencies above 100 Hz, it reaches a 6 fT/Hz^1/2^ level. Our improvement in sensitivity at low frequency can be essential, for example, in MEG applications, where the sensitivity on the order of 5 fT/Hz^1/2^ is a conventional level achieved with low-Tc SQUIDs.

**Figure 1:**
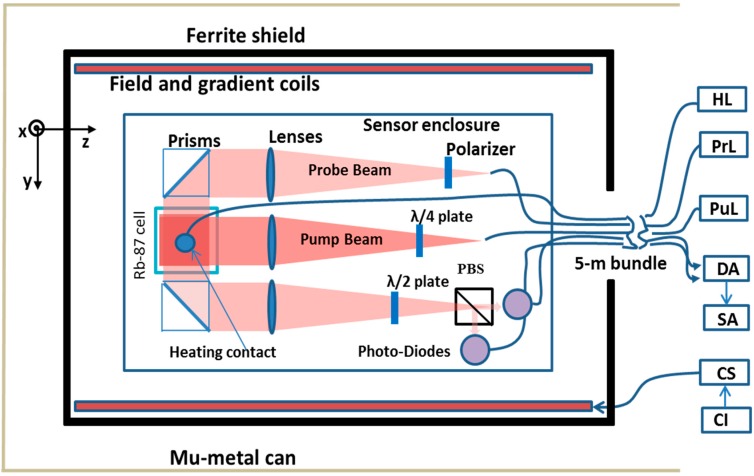
Diagram of fiber-coupled AM with laser heating: HL—Heating Laser, PrL—Probe Laser, PuL—Pump Laser, DA—Differential Amplifier, SA—Spectrum Analyzer, CS—Current Source, CI—computer interface.

Our AM (Figure 1) contains a 1 × 1 × 1 cm^3^ ^87^Rb cell filled with a nitrogen buffer gas at 600 Torr. The pump and probe beams are delivered to the atomic cell by polarization-maintaining (PM) fibers and are expanded with lenses to match the cell volume. The pump beam (λ = 795 nm) is circularly polarized with a λ/4 wave plate and orients the atomic spins in the direction of its propagation. The linearly polarized probe beam (detuned slightly from λ = 795 nm by ~100 GHz for the signal maximum response) is sent through the cell perpendicular to the pump beam, and its polarization rotation is measured with a balanced polarimeter consisting of a polarizing beam splitter (PBS) and two photodiodes. With this arrangement, the y-projection of the atomic spin is sensitively measured. A bias field is applied along the pump beam direction to tune the magnetometer’s maximum response to a given frequency. Laser heating from a JDS Uniphase corporation (JDSU) high-power (940 nm, *P_L_* = 0.85·(*I* − 0.5), where *P_L_* is the laser power in W, and *I* is current in A) fiber-coupled diode laser is arranged by attaching a multimode fiber to the cell with high-temperature cement (heating contact in Figure 1). The temperature of the cell was estimated from the probe-beam absorption measurements *T* = 50 + 16.5*P*, with the power required to achieve an operational temperature of 104 °C about 3.3 W. The AM head has dimensions 4.8 × 6.0 × 12.8 cm^3^ and is connected to electronics modules and lasers with a 5-m cable. The size of the sensor head is not optimal for MEG applications; however, it can be reduced with more careful design and by reducing the size of the cell, which would lead to a proportional reduction in all other elements. The reduction in the optical path length, that is the distance traversed by the probe beam inside the vapor cell, can be compensated by heating the cell to a higher temperature, which is automatically achieved for even smaller heating laser power.

## 3. Results

It is anticipated that the AM of this design can work in a wide range of frequencies with an appropriate tuning of the bias field. At very high frequencies of 23 kHz and 40 kHz, we have already shown that the AM of this design can reach 5 fT/Hz^1/2^ and 4 fT/Hz^1/2^ sensitivity [21]. Sensitivity is expected to be of a similar order even at much higher frequencies, since the width of the resonance curve does not depend much on the bias field after 40 kHz, but the noise in general is saturated by photon shot noise. However, it is not at all obvious that the AM can perform well at very low frequencies or between dc and 23 kHz. One problem with low frequency operation is that laser technical noise is much higher, especially due to the fibers used to deliver the light to the cell and feedback from the reflections. Technical noise of both the probe and pump lasers is important. While the optical rotation increases due to a smaller bandwidth, the signal-to-noise ratio depends on the ratio of the gain in the signal and the increase in the noise. The gain in the signal is expected to be about 16 from the ratio of magnetic resonance width (current measurement at low frequency gave half-width half-maximum (HWHM) of 18 Hz, while previously at high frequency, the width was 287 Hz). The increase of laser technical noise is anticipated inversely with the frequency (1/f noise), which starts to dominate photon shot noise below a few kHz. Thus by conducting measurements at very low frequencies, we can interpolate sensitivity in a large range of frequencies. We know that the SERF regime by definition is when the spin-exchange broadening can be neglected; hence, the magnetic resonance width is minimal. The increase in the width with the bias field is a well-known phenomenon and has been studied previously (see, for example, [15]). The spin-exchange broadening becomes significant at the bias fields corresponding to a few hundred Hz.

To test the AM noise at low frequency, the AM sensor head containing the cell was inserted into a cylindrical ferrite shield with end caps, which was placed inside a cylindrical mu-metal can. The magnetic noise suppression was on the order of 5000. The gradients and residual fields from the ferrite shield, which was in close proximity to the AM cell, were removed with a coil system wound on a cylindrical surface positioned inside the ferrite shield. Three orthogonal fields were generated with a solenoid and two orthogonal cosine coils; five independent first-order gradients, necessary at a low field to reduce the dominant gradients, were produced with a gradient coil system. The fields and gradients needed to maximize the AM signal were determined by scanning them using a Labview program.

The PM fibers were 5 m long in anticipation of applications outside the laboratory which would benefit from this distance from the electronics because of the reduction of noise and field distortions. Unfortunately, the longer fibers made the fiber-coupled DFB lasers, which did not have optical isolators, unstable. This caused the broadening of magnetic resonances due to light shifts arising from the circularly polarized pump beam. To improve stability, we increased the laser current and added an attenuator to reduce feedback from the long fiber, after which hop-free operation was restored.

The noise spectrum data were taken when the AM cell was heated to a temperature corresponding to Rb density of $n_{\mathrm{Rb}}$ = 7.4 × 10^12^ cm^−3^ as estimated from the probe beam absorption measurement [21]. The rotation of the polarization of the probe light, which is proportional to signal of the AM, is:

(1) $$\phi =\frac{1}{2}lr_{e}cfn_{\mathrm{Rb}}D(\nu )P_{x}$$

where $D(\nu )=\frac{\nu -\nu_{0}}{{\left(\nu -\nu_{0}\right)}^{2}+{\left(\Gamma_{L}/2\right)}^{2}}$ is the dispersion Lorentzian with HWHM $\Gamma_{L}$, ν is the laser frequency, and $\nu_{0}$ is frequency of the center of absorption of the *D*_1_ line; l is the optical pathlength, $r_{e}$ is the classical electron radius, c is the speed of light, f is the Rb *D*_1_ oscillator strength of D1 line of Rb, and $P_{y}$ is the y projection of Rb polarization.

**Figure 2:**
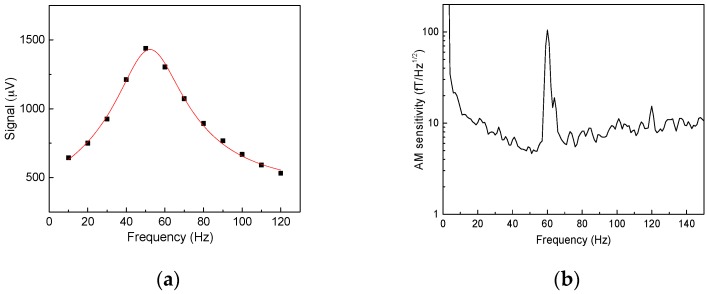
(**a**) The frequency profile of the AM response fit with Expression (2); (**b**) The sensitivity of the AM when it is tuned to 50 Hz with a bias field; the frequency response (**a**) is used to convert AM noise spectrum to sensitivity.

In the sensitivity demonstration for the dc-160 Hz range, the AM was tuned to the maximum response at 50 Hz with a $B_{z}$ bias field of 7.1 nT. The AM frequency response, obtained by applying a weak sinusoidal field whose frequency was changed in steps, was recorded and fitted with a Lorentzian plus a background line (Figure 2a):

(2) $$\frac{A}{\sqrt{{\left(\nu -\nu_{0}\right)}^{2}+\Gamma^{2}}}+B+C\nu$$

The line accounts for the contribution from the second peak at negative $\nu_{0}$ frequency [22,23,24] when the oscillating field rather than rotating field is applied. In-phase and out-of-phase components need to be considered; moreover, the magnetic field gradients and light-shift gradients can lead to deviation from the simple two-peak model, but these and many other effects, such as slow drifts in laser power and the AM signal, can still be approximated for our given resonance frequency, width, and range of frequencies at which the fitting was performed with a line: B+Cν. The HWHM Γ was found to be 18 ± 1 Hz, which is reasonable for the given Rb AM parameters. The width depends on bulk and wall collisions, the probe, pump spin-destruction, light shifts, and dephasing due to field non-uniformity, which was minimized by adjusting the first-order gradients. The theoretical estimate of Γ from Rb-Rb, Rb-N_2_, and the diffusion to the wall spin-destruction collisions is 8.3 Hz. Normally, the optimal pump rate in the SERF regime is equal to the spin-destruction rate, so the width is expected to be ~16.6 Hz, in agreement with the experiment. Further investigation on the optimal pumping rate at different bias fields will be given later.

The magnetic field sensitivity, which was rescaled by dividing the AM output noise spectrum by the AM frequency response, is presented in Figure 2b. The best sensitivity of 5 fT/Hz^1/2^ was achieved at 50 Hz, while a sensitivity greater than 10 fT/Hz^1/2^ was achieved in a frequency range from 15 to 150 Hz without an additional bias field tuning or a pumping rate adjustment of interest to MEG and MCG applications. It is possible to adjust the bias field to achieve high sensitivity in a much wider frequency range for applications such as ultra-low field NMR [10,11,12] and radio communication, including underground communication wherein there are limitations due to skin-depth penetration. In order to investigate the sensitivity in a wider range, we developed a model for the magnetic resonance width. This model is the further refinement on a vector model presented in [25], in which the total angular momenta ${\vec{F}}_{1}$ and ${\vec{F}}_{2}$ of lower and upper hyperfine manifolds precess in opposite directions in the magnetic field with equal angular velocity. Thus, the angle between the initial orientation and the orientation at time *t* for each component is $\varphi =\pm \omega_{0}t$. The precession frequency $\omega_{0}$ for each component is the precession frequency of the free electron divided by 2*I* + 1, the slowing down factor due to the presence of nuclear spin. When a spin-exchange SE collision takes place, the angular momenta are realigned and the relative change in the length of the sum of angular momenta $\vec{F}={\vec{F}}_{1}+{\vec{F}}_{2}$ of the two components can be found geometrically:

(3) $$\frac{\Delta F}{F}=1-\sqrt{\cos^{2}\varphi -{\left(\frac{F_{1}-F_{2}}{F_{1}+F_{2}}\right)}^{2}\sin^{2}\varphi }$$

Here $F_{i}=|{\vec{F}}_{i}|$. For a small φ, that is when the SE collision rate is higher than the precession frequency, the change in the angular momentum is close to zero, so no significant relaxation is taking place due to SE collisions—the SERF regime. The expansion for small angles φ proportional to the ratio of the field to the SE rate leads to a quadratic dependence on the field, which is well known for a small polarization case [15]. For a large φ, it is important to introduce statistical averaging, since the collision time is not a fixed value but a random function:

(4) $$\frac{\Delta F}{F}=\int_{0}^{\infty }\left[1-\sqrt{\cos^{2}\omega_{0}t-{\left(\frac{F_{1}-F_{2}}{F_{1}+F_{2}}\right)}^{2}\sin^{2}\omega_{0}t}\right]e^{-tR_{SE}^{\prime }}R_{SE}^{\prime }dt$$

Here, $R_{SE}^{\prime }$ is the rate of spin-exchange alignment of the total angular momenta of colliding atoms [25] (parallel directions of F_1_ and F_2_ after collisions), which is somewhat different from the SE rate $R_{SE}$ defined as the rate of reversal of electron spins. In spin-temperature approximation, the expression $\frac{F_{1}-F_{2}}{F_{1}+F_{2}}=2-4/\left(3+P^{2}\right)$ for I = 3/2, $3-48\left(1+P^{2}\right)/\left(19+26P^{2}+3P^{4}\right)$ for I = 5/2, or $4\left(1+7P^{2}+7P^{4}+P^{6}\right)/\left(11+35P^{2}+17P^{4}+P^{6}\right)$ for I = 7/2 [25] is a function of polarization P, so Equation (4) is a function of polarization, too:

(5) $$\frac{\Delta F}{F}=\int_{0}^{\infty }\left[1-\sqrt{\cos^{2}\omega_{0}t-{\left[2-4/\left(3+P^{2}\right)\right]}^{2}\sin^{2}\omega_{0}t}\right]e^{-R_{SE}^{\prime }t}R_{SE}^{\prime }dt$$

**Figure 3:**
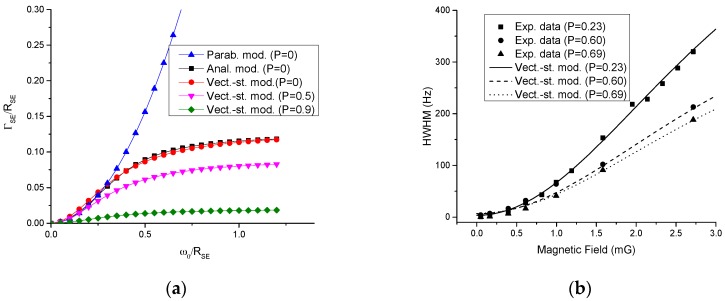
(**a**) The frequency profile of the width (spin-exchange part only, $\Gamma_{SE}$) using the prediction of the derived here vector-statistical model Equation (6) (*P* = 0, 0.5, 0.9) and analytical expression for *P* = 0 [26], which at small bias field can be approximated with a parabola [15]; (**b**) Comparison of a vector-statistical model and well controlled experimental measurements [25] for magnetic resonance width (this includes spin destruction contributions, Equation (7)) as the function of the bias field for different polarizations. Excellent agreement can be observed between the vector-statistical model and the experimental data. This model also reproduces results for the case of a large magnetic field and a high polarization: $2\pi \Gamma_{SE}=R_{SE}\left(1-P\right)/5$ derived for the radio-frequency (RF) magnetometer and used for finding the optimal pumping rate when the pumping rate term is added: $\Gamma =\frac{R_{SE}R_{SD}}{5R}+R/4$ [27].

Here, we consider in detail the case of I = 3/2; however, in the following equations, $\frac{F_{1}-F_{2}}{F_{1}+F_{2}}$ can be replaced with expressions for other nuclear spins. From the comparison with the analytical model Equation (99) in [26], we find that $R_{SE}^{\prime }\approx R_{SE}/1.5$. Furthermore, the accuracy of the model can be improved by multiplying it by a factor of 0.8 to make it closely agree with the analytical expression for zero polarization Equation (99) in [26] as illustrated in (Figure 3a):

(6) $$\frac{\Delta F}{F}=0.8\int_{0}^{\infty }\left[1-\sqrt{\cos^{2}(1.5\omega_{0}t/R_{SE})-{\left[2-4/\left(3+P^{2}\right)\right]}^{2}\sin^{2}(1.5\omega_{0}t/R_{SE})}\right]e^{-t}R_{SE}dt$$

**Figure 4:**
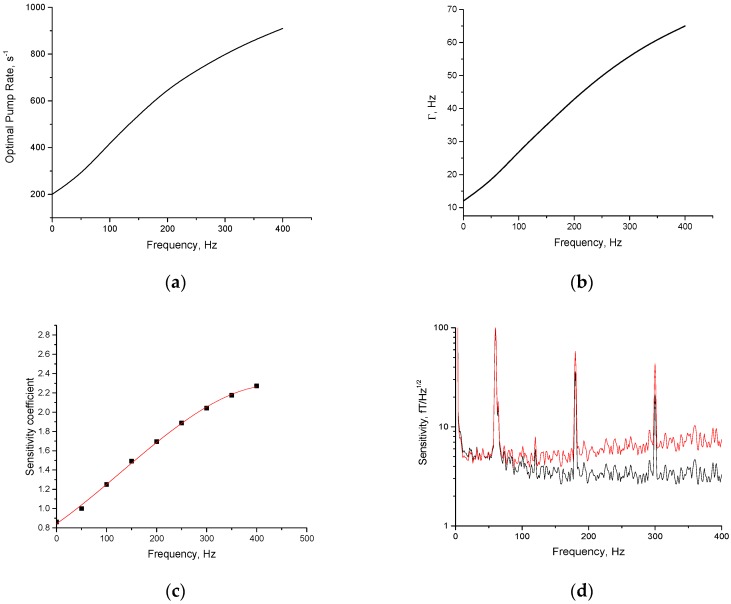
(**a**) The optimal pumping rate for different frequencies which maximizes the atomic magnetometer response and hence sensitivity limited by probe laser noise; (**b**) the magnetic resonance width (bandwidth of the magnetometer) for different frequencies to which bias field is tuned for the optimal pump rate given in (**a**); (**c**) the sensitivity coefficient which is the inverse of the AM response (proportional to $P_{x}\propto \frac{qR}{\left(R+R_{SD}\right)\Gamma }$ in Equation (1), where *q* and Γ are functions of frequency and optimal pumping rate, while the optimal pumping rate is also the function of frequency) to an oscillating field at the center of the magnetic resonance when the bias field is applied and the pump rate is optimized to maximize the response for frequencies in 50 Hz incremental steps; it is normalized to unity at 50 Hz; the fitted polynomial curve $f\left(\nu \right)=0.843+0.0036\nu +5.96\times 10^{-6}\nu^{2}-1.55\times 10^{-9}\nu^{3}$ for the sensitivity coefficient interpolates the data between the calculated points; (**d**) Sensitivity of AM (red curve) in 0–400 Hz range after multiplying the raw noise calibrated at 50 Hz (black curve) by the AM sensitivity coefficient, accounting for the width and polarization level variation with bias field and optimal pumping rate.

This analytical expression can be rewritten in terms of atomic magnetometer parameters and spin-destruction terms can be added to obtain the total broadening to compare with experiment:

(7) $$\Gamma =\frac{0.8}{2\pi }R_{SE}\int_{0}^{\infty }\left[1-\sqrt{\cos^{2}\left(k\nu_{expt}t/R_{SE}\right)-{\left[2-\frac{4}{3+{\left[R/\left(R+Rsd\right)\right]}^{2}}\right]}^{2}\sin^{2}\left(k\nu_{expt}t/R_{SE}\right)}\right]e^{-t}dt+\frac{R_{SD}}{q}+\frac{R}{q}$$

where R is the pumping rate, $R_{SD}$ is the spin-destruction rate, *q* is the slowing down factor, equal to 4 at high-field or high-polarization limits and to 6 at zero polarization, k=3πq/4, and $\nu_{expt}$ is the experimental Larmor frequency in Hz related to the bias field 2.8 B/q·MHz/G. The slowdown factor was used in [15] to relate the spin-destruction rate $T_{2}^{-1}$ due to atomic collisions to the observed HWHM SERF resonance in Hz: $\Gamma =\Delta \nu =1/2\pi qT_{2}^{-1}$. The slowing down effect is implicitly included into the SE term in Equation (7), but the spin-destruction and pumping broadening terms contain it explicitly. For arbitrary polarization, the slowdown factor is given analytically in [25] for the range of fields $\frac{\omega_{0}}{R_{SE}}<0.3$, which is sufficient for our specific conditions. Beyond this range, the slowing down factor is close to 4. The comparison of vector-statistical model in the form of Equation (7) (units of $\nu_{expt}$ are replaced with units of bias field according to $\nu_{expt}=$ 2.8 B/q·MHz/G) with the experimental data [25] in Figure 3b for different bias fields and polarizations shows excellent agreement, further validating our model. With the help of this model, we find the width of the resonance curve as a function of the pumping rate and the bias field and optimize the pumping rate to obtain the maximum response $S\propto qR/\left(R+R_{SD}\right)\Gamma$ for a given bias field. We tune the bias field so that the AM exhibits the resonance at a given frequency, in contrast to the case of Figure 2, where a single bias field was used corresponding to a 50 Hz resonance frequency. Figure 4a shows the optimal pumping rate for different frequencies; Figure 4b shows the corresponding bandwidth of the magnetometer when the pumping rate is optimized; Figure 4c shows the corresponding sensitivity coefficient that the dominant optical probe noise needs to be multiplied by to obtain the expected sensitivity at different frequencies for an experimental noise level (Figure 4d). Figure 4d illustrates the sensitivity in 0–400 Hz; beyond this range, the sensitivity coefficient gradually increases by 20%, reaching a plateau at 2 kHz. We expect a sensitivity of 10 fT/Hz^1/2^ or better, if noise falls off, since laser technical noise gradually decreases to the level of shot noise. In our previous work, we indeed observed 5 fT/Hz^1/2^ at 10 kHz, which is consistent with the current extrapolation if we also take the threefold higher density of vapor in [21] into account, and the scaling of the signal as square root of density when spin-destruction is not dominated by Rb-Rb collisions. Unfortunately, raising the temperature can lead to the burning of the cement and the fiber, so we used the temperature at which the magnetometer can work for a very long time. Further improvement in laser heating design can lead to a higher temperature of the cell and better sensitivity. We assumed throughout that the spin-exchange rate is 7760 $s^{-1}$, which corresponds to the Rb density 7.4 × 10^12^ cm^−3^ for the SE cross-section of 1.8 × 10^−14^ cm^2^. The theoretical spin destruction rate was also calculated from the Rb-Rb and Rb-N_2_ cross sections, and the diffusion to the walls [15], to be 206 $s^{-1}$, and this value was used in the modeling of the magnetic resonance curves of the magnetometer. 

In addition to the bandwidth, which is equal to the magnetic resonance width, it is important to characterize the magnetometer in terms of dynamic range, since it operates in the open-loop mode. According to [15], in the SERF regime and quasi-static approximation, the field at which the magnetometer response reaches maximum is $B_{m}=\frac{\Gamma }{g}$, where *g* is the gyromagnetic coefficient 700 × 10^7^ Hz/T; for  Γ= 18 Hz, this field is 2.6 nT resulting in the dynamic range of $B_{m}/dB=5.2\times 10^{5}$, where dB is the sensitivity level at 5 fT. In a non-SERF regime, the dynamic range is even higher due to a larger bandwidth (BW). The level at which the response is saturated can be found by solving the Bloch equation in the rotating frame. At resonance, $B_{m}=\frac{1}{g\sqrt{T_{1}T_{2}}}$. Because $T_{2}<T_{2,SERF}\approx T_{1}$ in the non-SERF regime, the dynamic range is larger. Some complications arise due to the additional amplitude-dependent SE broadening owing to the reduction in light narrowing and hence non-linear response [28].

## 4. Discussion

Compared with single-beam fiber-coupled magnetometers [4,5,6], the AM with the orthogonal pump-probe arrangement provides the following advantages: a higher ultimate sensitivity, an option for passive operation without modulation, and sensitive operation in a wide frequency range with corresponding bias field tuning. Passive operation can be necessary to avoid interference between channels in multi-channel systems or in situations, for example in NMR detection, when modulation can affect the measurements. The tunability in a wide range of frequencies from dc to very high frequencies is a great asset for applications such as underground radio-communication and NMR [10,11,12]/MRI [13,14]/NQR [29,30] detection. In many high-frequency applications, to avoid strong noise components, the frequency can be shifted to a quiet frequency range. Similar to one-beam magnetometers, the two-beam magnetometers will be valuable for MEG recordings. Passive operation in MEG that would eventually require hundreds of channels is an important option for the reduction of cross-talk. Our two-beam magnetometer can also be used in other applications, such as liquid explosive detection via ultra-low field MRI (airport scanners) at a frequency of 2 kHz [31], which is beyond the range of single-beam magnetometers [4,5,6]. A single beam configuration is inefficient at high frequency because the spins need to be tilted by the magnetic field to avoid a quadratically small signal response to the small measured field, but this decreases polarization level and hence reduces a light-narrowing effect.

Apart from the two-beam aspect of our AM, another important feature is laser-based heating. Previously, it was used with small 4 mm cells [5], but now we applied this technique to heat a much larger 1 cm cell. For this cell, we estimated that the temperature gradient was about 10 degrees, resulting in some density non-uniformity, which is an inverse function of absolute temperature, and in variation in the spin-destruction rate by less than 3%. A more important effect might be the non-uniformity of the pumping rate and the polarization level near walls. The study conducted in [32] showed that, despite various issues that can cause non-uniformity of the signal across the cell, the product of spin-density and spin-polarization did not vary much to negatively affect the sensitivity. Although electrical heating used in several atomic magnetometer demonstrations [4,5,6] is quite simple and efficient, it introduces some noise and can lead to interruptions in measurements. The laser-based heating does not interfere with magnetic measurements and allows continuous measurement. It can be particularly useful for setting future sensitivity records in small-cell magnetometers. Unfortunately, at present, laser heating in our AM was suboptimal for a relatively large cell and prevented us from reaching a temperature high enough to significantly improve the sensitivity of small cells. Still, our demonstrated sensitivity is comparable to that of other state-of-the-art AMs and SQUIDs. In some cases, our low-temperature magnetometer will have advantages when the object needs to be placed within a minimal distance from the sensor area (e.g., micro-fluidic remote NMR detection [33] and flux-concentrator enhancements in sensitivity [34,35]).

## 5. Conclusions

In summary, we demonstrated the high-sensitivity operation (5 fT/Hz^1/2^) of a two-beam compact fiber-couple atomic magnetometer at low frequency (50 Hz) and better than 10 fT/ Hz^1/2^ in a wide range of frequencies. To analyze magnetometer response outside SERF regime, a vector-statistical model was developed. Many applications are anticipated, especially in national security and MEG. The advantage of the current design is a passive operation (no electrical heating and no modulation field) and a broad-range tunable operation. Current sensitivity was limited by the temperature of the cell heated with laser light. With improvements in heating anticipated in the future, sub-fT sensitivity is expected. Another important point is that a five-meter cable containing optical fibers and electrical wires was implemented to allow us to remove electronics at this distance from the place of measurements. This will be important in applications outside a shield, in low-frequency communication, in NQR-based mine detection, and with geophysical surveys. Gradiometers can be built with better ambient noise suppression when the electronics are at a significant distance.
